# Problems of communication skills of GPs in Bulgaria

**DOI:** 10.1093/eurpub/ckac131.537

**Published:** 2022-10-25

**Authors:** B Vasileva, M Karcheva

**Affiliations:** 1 Department of Therapeutic Care, Faculty of Health Care, Medical University-Pleven, Pleven, Bulgaria; 2 Department of Epidemiology, Medical University-Pleven, Pleven, Bulgaria

## Abstract

**Problem:**

The effective communication between a doctor and a patient is considered to be essential, for providing high-quality medical care. A new model of cooperation and partnership which focuses on the patient is being established in the modern healthcare.

**Description of the problem:**

Data collected by empirical study on the doctor-patient communication has been presented and analyzed based on an overview analysis of the phenomenon communication. An anonymous survey among respondents was taken in the period September 1st-30th 2018 in the region of Pleven, Bulgaria. The respondents were patients who visited their GPs during that period. The results were processed with Microsoft Office Excel 2007. The quantitative analysis was done with statistical software programs - SPSS 17.0.

**Results:**

The respondents in the region of Pleven were 1053. It was found that the patients’ satisfaction was poor in the matter of two crucial issues: 481 (46 %) of the respondents were dissatisfied with the time that was spent for a conversation with each patient and 470 (45 %) - with the information given by the GP regarding their health condition. According to 345 of the respondents (32,8 %) GPs do not practice informed consent and it is rarely required from patients according to 317 of the respondents (30,3 %).

**Lessons:**

The analysis shows that there are a number of flaws and unsolved problems in the doctor-patient communication. The verbal communication of the doctor is a serious problem: the ability to ask and to listen. The need of communication training for the doctors in their gradiate study and postgraduate education is evident. It is recommended that a specific training programs for communication in the primary health care should be developed and evaluated.

**Key messages:**

• At the present time the communication skills of the general practitioner should be acknowledged and regulated as priority criteria for their overall assessment.

• It is necessary that a profound study on the barriers for effective communication between a doctor and a patient is conducted.

